# Fuel moisture content enhances nonadditive effects of plant mixtures on flammability and fire behavior

**DOI:** 10.1002/ece3.1628

**Published:** 2015-08-22

**Authors:** Luke G Blauw, Niki Wensink, Lisette Bakker, Richard S P van Logtestijn, Rien Aerts, Nadejda A Soudzilovskaia, J Hans C Cornelissen

**Affiliations:** 1 Systems Ecology, Department of Ecological Sciences, VU University AmsterdamAmsterdam, The Netherlands; 2 Conservation Biology Department, Institute of Environmental Sciences, Leiden UniversityLeiden, The Netherlands

**Keywords:** Combustion, heather, moss, plant traits, species composition, surface fuels

## Abstract

Fire behavior of plant mixtures includes a complex set of processes for which the interactive contributions of its drivers, such as plant identity and moisture, have not yet been unraveled fully. Plant flammability parameters of species mixtures can show substantial deviations of fire properties from those expected based on the component species when burnt alone; that is, there are nonadditive mixture effects. Here, we investigated how fuel moisture content affects nonadditive effects in fire behavior. We hypothesized that both the magnitude and variance of nonadditivity in flammability parameters are greater in moist than in dry fuel beds. We conducted a series of experimental burns in monocultures and 2-species mixtures with two ericaceous dwarf shrubs and two bryophyte species from temperate fire-prone heathlands. For a set of fire behavior parameters, we found that magnitude and variability of nonadditive effects are, on average, respectively 5.8 and 1.8 times larger in moist (30% MC) species mixtures compared to dry (10% MC) mixed fuel beds. In general, the moist mixtures caused negative nonadditive effects, but due to the larger variability these mixtures occasionally caused large positive nonadditive effects, while this did not occur in dry mixtures. Thus, at moister conditions, mixtures occasionally pass the moisture threshold for ignition and fire spread, which the monospecific fuel beds are unable to pass. We also show that the magnitude of nonadditivity is highly species dependent. Thus, contrary to common belief, the strong nonadditive effects in mixtures can cause higher fire occurrence at moister conditions. This new integration of surface fuel moisture and species interactions will help us to better understand fire behavior in the complexity of natural ecosystems.

## Introduction

The occurrence of fire in an ecosystem is a chance process and therefore hard to predict, but to approach accurate predictions all drivers of fire behavior should be examined, and their interactions unraveled. Wildfires can lead to a change in species composition, vegetation destruction, enhanced carbon emission and changing soil hydrology (Bowman et al. 2009; Stoof et al. 2010; van der Werf et al. 2010; Bernhardt et al. 2011; Turetsky et al. 2011). Therefore, several aspects of fire behavior have already been investigated, such as water dynamics and fuel ignition and combustion processes (Rothermel 1972; van Wagner 1977; Alexander 1982; Rothermel et al. 1986; Van Wilgen et al. 1990). Besides, abiotic factors, such as temperature and precipitation, play a considerable role, because together with the fuel properties they determine the fire behavior (Westerling et al. 2006; Kloster et al. 2010; Pausas and Ribeiro 2013).

The course of fire and the amount of carbon released partly depend on the different types of fuel, that is, living plant material, litter, and/or soil organic carbon (Schwilk 2003; Mack et al. 2011). The properties of these fuels determine the fire properties, because fuel types differ in their water dynamics, chemistry, and structure, which cause differences in flammability (Melillo et al. 1989; Krankina and Harmon 1995; Schlesinger and Andrews 2000). The water dynamics are dependent on the species composition in a system, because at individual species level there are differences in water management (Ellison et al. 2005). The composition and abundance distribution of species influence the water balance of the whole system. In turn, the system's water balance, indirectly via drought, has a large effect on the fire regime of that system (Swetnam and Baisan 1996; Dale et al. 2001; Bradstock 2010). The structure and particle size distribution indirectly influence flammability via the interaction between fuel surface and oxygen (Burrows 2001). This property differs among species and is, together with moisture content, important in fire behavior (Bond and Van Wilgen 1996; Cornelissen et al. 2003; Alessio et al. 2008a,b; Schwilk and Caprio 2011; Cornwell et al. 2015). For a given species, the moisture content and plant structure interact and indirectly influence the fire behavior (Zhao et al. 2014).

In the case of living plant parts and the litter derived from them, the structural, chemical, and moisture-related fuel properties, commonly called plant traits, together determine flammability (Bond and Midgley 1995; Cornelissen et al. 2003; Scarff and Westoby 2006; Grootemaat et al. 2015). The effects of variation in traits on initial fire behavior have already been investigated extensively among single species (Pinard and Huffman 1997; Schwilk 2003; Pausas et al. 2004; Schwilk and Caprio 2011). Several studies found, for instance, that the ability of a plant material to retain moisture has a large influence on its flammability (Rothermel 1972; Burrows 2001; Alessio et al. 2008b; Zhao et al. 2014). Leaf size is another key trait for flammability via its positive effect on internal fuel bed aeration through looseness of packing (Scarff and Westoby 2006; Cornwell et al. 2015). Although these and further studies have shown clear relations between plant traits and flammability, almost all of them are based on monospecific surface fuel beds, which is relevant for forest plantation or other monospecific stands but not representative for most natural ecosystems.

Plant flammability studies on the effect of species interactions in laboratory setups, through the interaction between traits of different species, address a relatively new scale to examine fire behavior. This contrasts with other fields of research, such as litter decomposition rates and vegetation productivity or crop yield, where the trait interactions between species have already been shown to cause larger (or sometimes smaller) effects than expected based on the component species when decomposed or grown alone, the so-called nonadditive effects (Cardinale et al. 2003; Smith and Bradford 2003). These findings raise doubts about model predictions based on species monocultures, which may be biased when, for instance, averages of multiple species are used. Recently, the interaction between species traits has been found to cause nonadditive effects on fire behavior of surface fuel beds (van Altena et al. 2012; de Magalhaes and Schwilk 2012). These studies found that nonadditivity in flammability parameters, for example, ignitability, rate of fire spread, and heat release, can occur as a result of both mixing different plant parts (e.g., leaves and twigs) and mixing the same plant part, in this case leaves, from different species (van Altena et al. 2012; de Magalhaes and Schwilk 2012). The fact that mixing different plant parts led to nonadditive effects could suggest that species mixtures composed of different plant functional types (PFT), thus having contrasting traits (Chapin et al. 1996; Diaz and Cabido 1997), show stronger nonadditivity. The interaction between species with different internal fuel density and particle size, through its consequences for surface fuel bed configuration and energy content, is probably one of the main drivers of these nonadditive effects.

In summary, moisture content and water retention capacity of plant material in surface fuel beds, as well as structural traits such as particle size, are important drivers of plant flammability. Moreover, plant flammability parameters of fuel mixtures cannot simply be predicted from the average flammabilities of the component species when burnt alone. For example, the ICFME model translates measured plant properties into a single component layer, which could be one of the reasons that the fire behavior of collected data does not match with the model predictions (Butler et al. 2004; Alexander and Cruz 2013). Together these points raise the intriguing and important question whether and how nonadditivity of flammability in plant mixtures depends on surface fuel moisture. More specifically, our study addresses the following questions: (1) Does moisture content determine the magnitude of nonadditivity of plant mixtures' flammability? (2) Does the magnitude of nonadditivity among mixtures vary in consistent and predictable ways? We hypothesize (1) that nonadditive mixture effects on flammability variables will be greater and more variable at higher moisture contents, which are likely close to the threshold moisture for fuel ignition of some plant materials. Among mixtures, we expect (2) differences in the magnitude of nonadditivity, with larger effects in mixtures containing two different plant functional types (PFT).

To test these hypotheses, we first determined the effect of surface fuel moisture content (10% vs. 30% MC) on flammability of four species in monoculture, to quantify their individual flammability parameters. Then, we determined the effect of variation in moisture content on plant mixture flammability of all possible combinations of species pairs. We used four species predominant in fire-prone temperate heathlands, belonging to two groups that are functionally distinct and phylogenetically distant: the evergreen ericaceous dwarf shrubs *Empetrum nigrum* (L.) and *Calluna vulgaris* (L.) Hull and the moss species *Pleurozium schreberi* (Brid.) Mitt. and *Hypnum jutlandicum* Holmen & Warncke.

## Methods

### Study site and sampling

Four dominant northwest European heathland species, from two distinct clades and plant functional types, were collected from two dry heathland locations at the Veluwe, in the central part of the Netherlands (52°09 N, 5°64 E and 52°01 N, 5°93 E) at the end of October 2010. In the Netherlands, a temperate climate prevails with an average annual temperature of 10.1°C and precipitation of 833 mm (KNMI 2010). Currently, such heathlands have low but significant fire regimes mainly in early spring and late summer; current climate change predictions include increased frequency and extent of drought periods, and thereby fire regimes (KNMI'14 scenarios, based on IPCC, 2013). The two pleurocarpous moss species, *Hypnum jutlandicum* and *Pleurozium schreberi*, generally occupy the bottom layer below the vascular plants. They are ectohydric, so, after an extensive dry period, they are very dry and can play a considerable role in fire. *Empetrum nigrum* and *Calluna vulgaris* are the two predominant vascular plant species in higher and drier parts of the heathland and play an important role in heathland fire regimes (Hobbs and Gimingham 1984; Davies and Legg 2011). From the selected species, the aboveground (partly green) plant material from multiple random individuals was collected, which in case of the heathers included the main stem with branches, leaves, and old seed heads.

### Treatment

**Figure 1:**
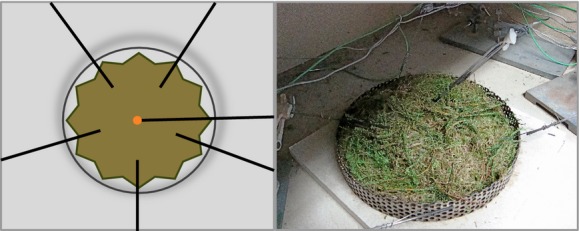
Schematic design and true image of the fire experiment fuel bed; the schematic design includes fuel, thermocouples, and the position of ignition.

All material was carefully cleaned of soil particles, litter, and other matter. To preserve natural fuel properties and density as much as possible, only pieces larger than 25 cm were cut smaller to fit the material into the fire ring of our experimental setup (see below and Fig.1). Species samples consisted of at least four individuals to create random and similar replicates for each species (Scarff and Westoby 2006). We adopted two types of treatments: species composition and moisture content. The species composition treatment consisted of all four single species and all six possible combinations of two species mixtures. These 10 compositions were burned at air-dried condition, equivalent to approximately 10% relative moisture content, and at a fuel moisture content (MC) of 30%. An air-dry subsample was measured for moisture content, and based on this content, water was added to the main sample, in a sealed plastic bag, to reach 10% or 30% relative MC (gravimetric sampling method). The plant material was thereafter incubated for 48 hours and opened (and mixed) only shortly before the burning experiment. At that time, subsamples were measured for actual percent MC (mean ± SD; 9.9 ± 1.8% and 28.8 ± 3.1%, respectively).

### Fire experiment preparations

The experimental burns were performed in the Fire Laboratory Amsterdam for Research in Ecology (FLARE) at VU University, Amsterdam (for details see van Altena et al. 2012). The fuel beds were created in a metal ring of 25 cm in diameter and 3 cm in height, perforated to ensure air exchange. This ring was placed on a solid fire-resistant plate under a fume hood that provided ventilation with a constant moderate air flow. A heater inside the room maintained a constant room temperature (18 ± 2°C) and air humidity (20–40%). The ring was filled with a volume of approximately 1.5 L of plant material, and in case of a mixed composition, a volume of 0.75 L for each species. We chose volume-based fire experiments, because the lateral spread is crucial and this way species provide equal contributions in fuel bed structure and its associated indirect effect on oxygen supply (van Altena et al. 2012). The plant material from both species was randomly mixed to fill the entire ring with only very gentle pressure to create a rather natural structure. The material was fully mixed to focus on species interactions and avoid an increasing number of possible fuel bed structures that would emerge in a layered composition. We separately tested for the effect on fuel configuration in a subset of two species and found both differences and nondifferences in fire behavior depending on the fire variable measured (Figure S3). In further research, this aspect of fuel bed structure on fire behavior is worth in-depth investigation. The material (per species) was weighed just before and after the experiment to determine the amount of material burned. Subsequently, six thermocouples (1-mm-thick type K thermocouple; TC Direct, Uxbridge, UK) were installed at 1 cm above the fire ring/fuel bed at different positions, measuring the fire temperature in the ring. The first thermocouple was located in the center, and the other five were equally distributed at 6.25 cm from the center. After preparations, a cotton disk was injected with 1 ml of ethanol (96%), positioned in the center of the fuel bed, and ignited using a lighter (Fig.1).

### Fire experiment

One burn for each treatment (single species or mixture of a given species composition at 10% or 30% MC) was performed weekly to create a block design that represents the replicates over a five-week period. Each experiment started with the ignition of a cotton disk, and simultaneously, the computer started registering the thermocouples' temperature. The fire then spread toward the edge of the ring, except for some cases where the fuel moisture content extinguished the fire immediately. During each burn, the room temperature and humidity were registered. The temperature recording was stopped after the last thermocouple had dropped below 50°C.

### Data analysis

As the initial flame from the cotton disk affects the thermocouple located in the center, only the data from the outer five thermocouples were used to determine the following parameters:

1. Rate of fire spread (cm/min) was calculated by dividing 6.25 cm (distance between middle and outer thermocouples) by the time period from the moment when the temperature at the central thermocouple reached 50°C to the moment when the fire reached the first outer thermocouple (Davies and Legg 2011; van Altena et al. 2012).
2. Maximum temperature was measured among all outer thermocouples. The central thermocouple was excluded, because the cotton disk might have caused the highest temperature (van Altena et al. 2012; Pausas et al. 2012).
3. Percentage mass loss (ML_%_) was calculated using the following formula:

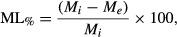
where *M*_*i*_ is the initial mass and *M*_*e*_ the end mass of the material after fire extinction (Ormeno et al. 2009; de Magalhaes and Schwilk 2012).

Next, the flammability measures (1,2,3) were compared between the different treatments in terms of mixed versus monospecific fuel beds and moisture content. To test our hypotheses, the expected values for mixtures were calculated as the average of two single species, and the effect size (Hedges and Olkin 1985; Hedges et al. 1999) of nonadditivity of the mixture was calculated as:

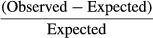


If the calculated values are not significantly different from 0, this means that the observed flammability of the mixture fuel bed does not deviate from the flammability that would be expected based on the average for two single-species fuel beds, that is, additivity. If the value is negative, this means that expected values are higher than observed, and thus, the two species, or one of them, have a negative influence on their joint flammability. If the value is positive, this implies the opposite, so the two species together enhance the flammability compared to their monospecific fuel beds. For both negative and positive nonadditivity, the value itself indicates the *relative* strength of the nonadditive effect.

In the above-described analyses, the species mixing was performed based on species volume, that is, the fire experiment, and the mixture fuel beds were filled based on plant volume. However this created differences in plant mass among species. Therefore, we also calculated their expected flammability based on the relative plant mass contributed by each species (van Altena et al. 2012). The mass ratio of each species in the fuel bed was multiplied with their single fuel bed flammability, and together, these values formed the expected flammability in a mass-based approach (Figure S1). Subsequently, a set of statistical analyses were performed, using R version 3.1.2. (R Core Team 2014), to answer our specified research questions.

### Statistical analyses

First, we compared the weekly performed fire experiments (i.e., blocks), using a one-way ANOVA, to determine whether the environmental conditions were similar among weeks. Prior to this, we verified with a Levene test that the data showed a normal distribution and that none of the flammability measures were significantly different among weeks, making each weekly burn a true replicate. After calculating the (non)additivity for each burning, the data did not show a normal distribution and contained five or less replicates per specific treatment. Therefore, we performed a ranked statistical analysis and compared the group medians by first ranking the data.

Second, we performed a Wilcoxon rank test to determine whether the mixtures in each of the moisture treatments caused nonadditive effects. In this analysis, the median and variance of all mixtures within a moisture treatment were compared to zero to test against the null hypothesis of additivity. If the median was significantly different from zero, the null hypothesis was rejected and the alternative hypothesis, that is, the presence of nonadditive effects, was accepted. Third, we performed a Bartlett test of homogeneity of variance to determine whether the distribution of nonadditive effects was different between the two moisture contents and among mixtures differing in species composition. This is because we expected the variability in nonadditive effects among replicates of a given mixture to be larger at 30% than at 10% moisture content (MC), reflecting the fact that 30% MC would be close to the threshold for ignition for at least some of the species (hypothesis 1). Usually a Bartlett test determines whether groups show equal variances as a condition for carrying out ANOVA, but our aim was the opposite and focused on variance in nonadditivity. Significant heterogeneity of variances would thus indicate that the moisture content and/or mixture caused a different range of nonadditivity, which would be in agreement with our first hypothesis. In addition, we performed a Kruskal–Wallis rank sum test to determine whether 30% MC caused larger nonadditive effects overall compared to 10%, because the distribution of variances would not prove a difference in means (ranked medians). To visualize the direction of nonadditivity, we created boxplot figures including both moisture content treatments. Last, a two-way ANOVA on ranked data was performed to test the nonadditivity between mixtures of different species compositions and to see whether the interaction between moisture and mixture species composition had an effect on the nonadditivity. Note that the factor moisture content was also statistically tested in the previous Kruskal–Wallis rank sum test and should show similar results, despite the minor differences in methodology. Prior to performing a two-way ANOVA, we performed a Levene test on the ranked data, to determine whether the data showed a normal distribution. If the two-way ANOVA showed significant results for mixtures or the interaction between mixtures and moisture content, a Tukey HSD post hoc test was performed to compare specific mixture pairs differing in species composition (cf. hypothesis 2).

## Results

**Figure 2:**
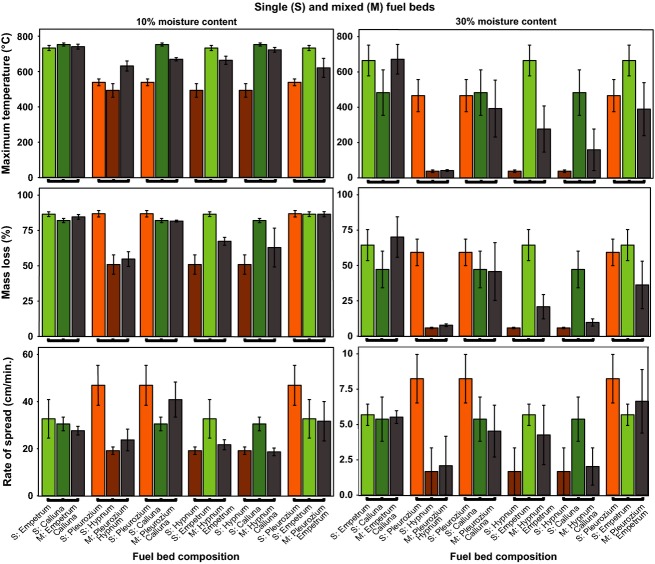
The maximum temperature, mass loss, and rate of spread for each monospecific and associated mixed fuel bed at both 10% (left panel) and 30% (right panel) moisture. The bars represent the average ± SE of all replicates for each fuel bed composition.

We found a larger variability in maximum temperature, fire spread rate, and (somewhat less so) percentage mass loss among high-moisture fuel beds compared to that in low-moisture fuel beds (Fig.2). Interestingly, within the moss species subset, *Hypnum jutlandicum was* in general less flammable compared to *Pleurozium schreberi* (brown colored bars vs. orange bars in Fig.2).

### Moisture content and the magnitude of nonadditivity

**Figure 3:**
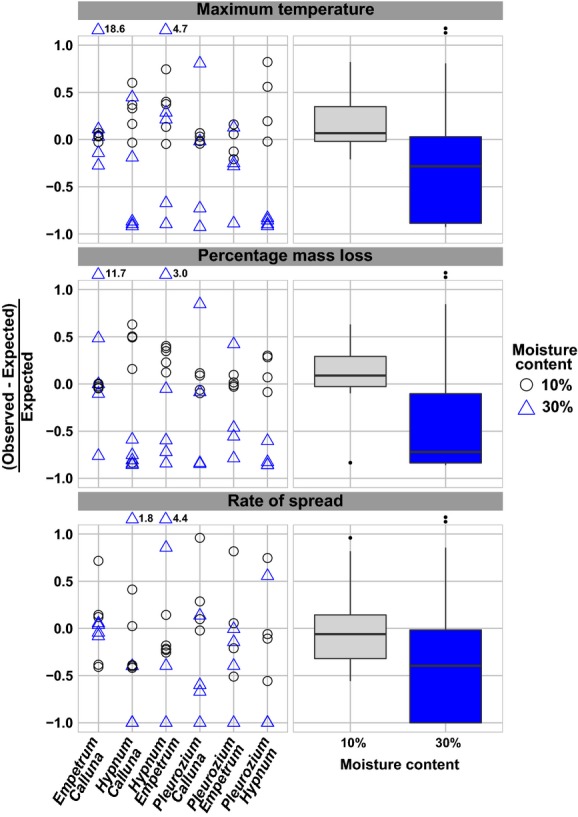
Effect sizes of (non)additive effects for different species mixtures at two moisture contents for three fire parameters adopting a volume-based approach. Left panel: (non)additive effects individually for each burn. Right panel: boxplots of all mixture values within a moisture content to show the grouped direction and magnitude of nonadditivity. Two extreme values per flammability parameter lie outside the *y*-axis limits, and their values are indicated.

We found strongly varying and often substantial nonadditive effects in plant mixtures in both the 10% and 30% moisture content (MC) treatment with respect to maximum fire temperature and percentage mass loss (Table1; Figs.2, 3 right panel). In contrast, for rate of fire spread, we found only additive effects. It should be stressed that nonburning fuel beds were given a spread rate of 0, which caused ties in the Wilcoxon rank test. In that case, the *P*-value could not be computed exactly and this led to the unexpected result that rate of fire spread showed no nonadditive effects in mixtures with 30% MC (Fig.3 right panel). More surprising is the fact that mixtures with 10% MC showed a positive nonadditive effect and the 30% MC mixtures showed a negative nonadditive effect overall (Fig.3, Table1).

**Table 1:** Multiple analyses of effects of moisture content and species composition of mixtures on three flammability parameters for the volume-based approach

Independent variable	Maximum flame temperature	Percentage mass loss	Spread rate
*Nonadditivity*			
10%^A^	*V* = 315**	*V* = 287*	*V* = 171
30%^A^	*V* = 107*	*V* = 86*	*V* = 84
*Variance*			
10–30%^B^	*K*^2^_(1)_ = 100.4***	*K*^2^_(1)_ = 76.3***	*K*^2^_(1)_ = 23.0***
10% mixtures^B^	*K*^2^_(5)_ = 18.5**	*K*^2^_(5)_ = 35.0***	*K*^2^_(5)_ = 4.88
30% mixtures^B^	*K*^2^_(5)_ = 56.4***	*K*^2^_(5)_ = 50.0***	*K*^2^_(5)_ = 23.7***
10% – one vs. two PFTs	*K*^2^_(1)_ = 0.3	*K*^2^_(1)_ = 5.4*	*K*^2^_(1)_ = 0.2
30% – one vs. two PFTs	*K*^2^_(1)_ = 26.2***	*K*^2^_(1)_ = 22.5***	*K*^2^_(1)_ = 4.9*
*Nonadditivity*			
10% vs. 30%^C^	*χ*^2^_(1)_ = 9.32**	*χ*^2^_(1)_ = 12.9 ***	*χ*^2^_(1)_ = 3.64
*Nonadditivity (interactions)*			
10%–30%^D^	*F*_(1)_ = 12.3**	*F*_(1)_ = 19.2***	*F*_(1)_ = 3.57
Mixtures*	*F*_(5)_ = 1.13	*F*_(5)_ = 1.03	*F*_(5)_ = 0.62
Plant function type^D^	*F*_(1)_ = 0.04	*F*_(1)_ = 0.07	*F*_(1)_ = 0.07
Moisture*Mixtures^D^	*F*_(5)_ = 1.97	*F*_(5)_ = 2.52*	*F*_(5)_ = 0.995
Moisture*Plant functional type^D^	*F*_(1)_ = 0.15	*F*_(1)_ = 0.77	*F*_(1)_ = 0.03

A: Wilcoxon rank sum test.

B: Bartlett's test of homogeneity of variance.

C: Kruskal–Wallis rank sum test.

D: Two-way analysis of variance.

*P*-values: ^*^P <* *0.05; ^**^P <* *0.01; ^***^P <* *0.001.

This table shows the results of the statistical analyses that test the null hypothesis (additivity)^A^, variance^B^, and differences^C^^,^^D^ in the (non)additivity between moisture contents or among mixture compositions within a moisture content treatment for different flammability measures. The data are either original^A,B,C^ or ranked prior to the analyses^D^.

For all flammability measures, the Bartlett test showed a significant difference in variances (distribution of (non)additivity values) between 10% and 30% MC, indicating that moisture content did indeed significantly affect the effect size distribution of nonadditivity: at a higher MC, the variance increased, while at 10% MC, the data points were more centered (Fig.3, Table1).

### Variability of nonadditivity among species mixtures

When we compared the variance among mixtures of different species compositions within specific moisture content treatments, the variance in both maximum fire temperature and percentage mass loss was different among mixture compositions in the 10% moisture content treatment. This indicates that certain mixture compositions caused a larger range of nonadditive effects than others. This is clearly shown by, for example, the small variance of nonadditive effects in the *Empetrum/Calluna* mixture compared to the large range of nonadditive effects that *Hypnum* caused in all mixtures it participated in.

Due to the non-normal data distribution, data were analyzed in different ways (see Methods). The results of the Kruskal–Wallis rank sum test showed that 30% MC caused larger nonadditive effects in the negative direction for maximum temperature and percentage fuel mass loss than 10% MC (Table1; Fig.3). Next, the ranked two-way ANOVA results showed that 30% MC caused larger nonadditive effects in the negative direction than 10% MC, confirming the Kruskal–Wallis test results. Furthermore, this analysis did not show differences in nonadditivity among mixtures of different compositions. In other words, the variance in nonadditive effects was different between mixtures, but the medians were not significantly different. The interaction between moisture content and mixture composition did have a significant effect on the nonadditivity of percentage mass loss. A Tukey HSD post hoc performed on this subset showed several specific significantly different pairs. Although most of these significant differences between MC treatment were between different species mixtures, one of them was between the 10% and 30% moisture content treatments of the mixture *Hypnum–Calluna* (*P* = 0.028). Moreover, the variance of mixtures with two distinct plant functional types was larger than that of mixtures with only one PFT. In 10% MC fuel beds, this was only the case for the mass loss parameter, whereas for the 30% MC fuel beds the variance was different for all fire parameters (Table1).

The mass-based analysis showed similar results, with only some minor difference in statistical output, but it did not change any of the conclusions drawn from the volume-based analysis (see Table S1 and Figure S1). Interestingly, the extreme nonadditive values changed among specific species pairs, confirming that species interaction causes nonadditive effect independent of the method chosen.

## Discussion

Our results show that species interactions in surface fuel beds can cause substantial nonadditive effects on fire behavior (relative to expectations based on monospecific fuel beds) and that such nonadditivity is larger in magnitude and more variable when the fuel contains a higher moisture level. Together with the findings of nonadditive effects by other studies in this field of research, it is clear that nonadditivity plays an important role in plant flammability, with effect sizes far outweighing those observed in studies on nonadditivity in decomposition or productivity (Cardinale et al. 2003; Smith and Bradford 2003; van Altena et al. 2012; de Magalhaes and Schwilk 2012). Model studies already showed that the interaction between fuel moisture and fuel interactions can affect the fire behavior in grasslands systems (McGranahan et al. 2012, 2013). Our study is the first to experimentally show that moisture content affects both the magnitude and variance of the nonadditive effects of species mixtures for several flammability parameters. This interaction provides a new insight in the underlying factors that determine fire behavior and complement model studies addressing the role of fuel interactions in fire regimes (McGranahan et al. 2012, 2013).

### Moisture as driver of nonadditivity in fire behavior

**Figure 4:**
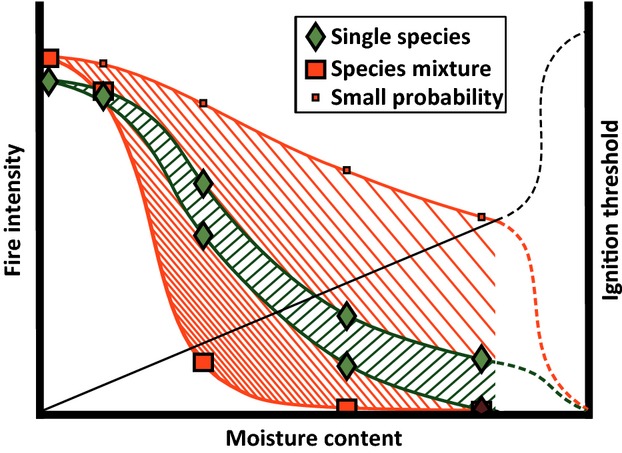
Conceptual representation of the effect of moisture on the fire intensity of single species and mixtures of the same species. The straight black line represents the hypothesized ignition threshold. The dashed lines represent speculative projections beyond 30% moisture content, that is, outside the boundary of this study.

Fire behavior of single species, or the average of two single species, is assumed to extinguish under wet conditions, but our experiment has demonstrated that a mixture of species can occasionally burn under these conditions. This is counterintuitive, but can be explained by the increase in variability in fire behavior of plant mixtures close to the threshold for ignition and fire development, which in single-species fuel beds lies generally between 20% and 30% MC (see Figure S2). This means that individual fuel beds occasionally pass this threshold and will still burn in spite of their substantial moisture content. We conceptualize this intriguing and previously unknown phenomenon in Figure4.

As is to be expected, under wet conditions, like in our flammable moist mixtures, fires occur only relatively rarely. This phenomenon complicates reliable fire predictions about moist mixed vegetation (see Table S2 for the effects of moisture on single-species flammability). Fire predictions should consider and anticipate the interaction between moisture and plant fuel mixtures, because especially near the moisture threshold the interactions between species can play a crucial role in fire behavior (Davies and Legg 2011).

The role of different species, through their morphological or chemical traits, is expected to have a great influence on the magnitude of nonadditivity in the mixture. To what extent each species contributes to the fire behavior may partly depend on the species it is mixed with. It could be that both species in the mixture contribute equally to the fire behavior, or it could be that the fire properties are based on one dominant flammable species in the mixture, “enhanced dominance” (van Altena et al. 2012). Our results (Fig.3) showed differences in nonadditivity between species pairs. Therefore, it is almost certain that moisture content is not the only driver of nonadditivity and that it is most likely a combination of other traits, for instance structural and chemical traits, that explain the magnitude and presence of nonadditivity (Eviner and Chapin 2003). The magnitude of nonadditive flammability may partly be determined by the differences in species chemical traits, especially when fuel bed configuration is not a factor (Philpot 1970; Rundel 1981; Dimitrakopoulos and Panov 2001; Alessio et al. 2008b; Pausas et al. 2012). However, the effects of chemistry on fire behavior are expected to have only minor influence when fuel structure is also a player (Lippincott 2000; Brooks et al. 2004). Other important traits that have already shown to be determinants of fire behavior are size, shape, and arrangement of plant material (Schwilk and Ackerly 2001; Scarff and Westoby 2006). These traits can indirectly, via fuel bed configuration and aeration, influence the fire behavior. In fully mixed, compared to layered, fuel beds, small particles can fill up spaces between the larger particles and thereby inhibit aeration of the fire, which may be especially important at a tipping point close to 30% MC (see Table S2 and Figure S2). On the positive side of nonadditivity in mixtures at 30% MC, a more ignitable species with low caloric content (e.g., *Pleurozium schreberi*) may help a less ignitable species (e.g., *Calluna vulgaris*) to overcome the ignition threshold, after which the latter, more calorie-rich species will help to sustain the fire. Our results already suggest a role for species identity (e.g., range of variability in *E. nigrum* vs. *H. jutlandicum* mixtures), but the exact role of traits and fuel bed structure is yet unclear. On the other hand, there is clearly a role for moisture in causing differences between species pairs. As Table S2 shows for the effect of moisture content on single-species flammability, the tipping point for fuel moisture content is between 20% and 30% MC. At 30%, the variability in fire behavior increases substantially, and for *Hypnum jutlandicum*, flammability already decreased substantially at 20% fuel MC. Interactions between species with different fuel moisture tipping points are probably the main cause for the variability. For example, with two species, one with a tipping point below 30% and one above 30% MC, the relative abundance of fuel near the ignition point may have determined the overall fire behavior. If the easily flammable species, even below 30% MC, had been relatively more abundant near the ignition source, then the fire could have ignited and developed. If the nonflammable species at 30% MC had been relatively more abundant near the ignition source, the fire could have stopped shortly after ignition. The fuel structure and configuration of different species in the mixture therefore could play an important role.

We did not find support for our second hypothesis, that is, pairs of species belonging to different functional types do not show consistently stronger nonadditivity than pairs of functionally more similar species, but we did find a difference in variance. Nevertheless, our results do show that under dry conditions the effects of species interactions on flammability are small and close to the average of the two monospecific fuel beds, while at moist conditions the variability generally increases and unlikely fire events may occur as a result of species interactions.

### Implications for wildfires and managements

We realize that under extremely dry conditions, when many organic fuels will burn easily, nonadditivity may only play a minor role in fire behavior. Therefore, we stress that the effect of nonadditivity on species flammability, as our results indicate, becomes particularly relevant when the weather condition becomes moist. In that scenario, species interactions start to play a more prominent role and can lead to fires under circumstances where they would not be expected based on knowledge about individual fuel types. In fire ecology, we tend to determine predictions based on average or median fire behavior, without specific focus on the variance around mean or median fire parameters. However, the temporal and spatial variability are typical elements that make fires difficult to predict (Kloster et al. 2010; McKenzie et al. 2011). In most fire prediction models, the species are represented by a static amount of fuel with certain properties (Rothermel 1972; Burgan and Rothermel 1984; Vanwilgen et al. 1985; Andrews 1986). The reliability of these models could substantially increase if not only the fuel load and moisture content are considered, but also the interaction between the species at various moisture levels. If model predictions lack interactions between essential drivers of fire, they are susceptible to produce mismatching predictions between the model and real scenarios. The frequency of these mismatches has caused the credibility of fire models to be challenged, also because the validation of these models before use has been lacking (Alexander and Cruz 2013). To improve the model prediction, the moisture threshold level for fire, which is a common parameter in fire prediction models, could take into account the number, (functional) identity, and abundance of species in the ecosystem (Rothermel et al. 1986; Kloster et al. 2010). By taking the variability of nonadditivity into account, the model predictions about fire behavior will better capture the variance in the processes involved. In this research field, great challenges ahead include quantifying the actual traits that are central in species mixing and moisture interactions on fire behavior, which would open the door for generalizing beyond individual species pairs. Also, the proportions and physical configuration of species mixtures had to be standardized to test the concept in our study, but will need to be varied to still better mimic those in the real ecosystems in which the species co-occur.

Another illustration of the significance of the interaction between moisture content and nonadditivity is in the field of fire regimes and species invasion. As discussed previously, the magnitude of nonadditivity is species dependent and this could be important when species invasion is considered (Dantonio and Vitousek 1992; McGranahan et al. 2013). When an invasive flammable species, which is also likely to interact with other species, establishes in an ecosystem, it can change the fire regime. For example, invasion by a flammable grass species may increase the fire frequency (Grigulis et al. 2005). As a consequence of the short recovery periods, woody vegetation, for example, may be unable to recover and the system therefore may change into a grass dominated system. The field study of Rossiter et al. (2003) and the model of Brooks et al. (2004) have both tested this theory and found that the ecosystem's fire frequency and intensity could change considerably through grass invasion. In these studies, little attention was paid to the underlying mechanisms, including plant traits and other factors and interactions that may determine the success of a species in affecting the fire regime. Based on our findings, nonadditive effects caused by the interaction between native and invasive species could play a role in the effects on the fire regime, and the strength and variance of such interactions may be influenced by environmental moisture.

## Conclusion

We have demonstrated that the magnitude and variance of nonadditivity depend on moisture content as the strongest determinant, with an additional key role for species identities. High moisture contents generally increase the nonadditive effects in the negative direction, but occasionally, in individual cases, also lead to strong positive nonadditivity. Such interactions add to fire's complex behavior. Nevertheless, this new element to species interactions on surface fuel flammability improves our understanding of fire behavior and could be used to improve fire models. In particular, when the effect of invasive species on fire regimes is evaluated, then nonadditive effects on flammability at different moisture regimes can be important to consider.

While our study has added a new and important element in fire behavior, field manipulation experiments are necessary to assess the relevance of our results in real ecosystems subject to current or future climatic conditions. Ecosystem invasion by a flammable species under different moisture content treatments would be a plausible setup to determine the interaction between moisture and nonadditivity on ecosystem fire regimes.

## Supporting Information

**Figure S1.** Effect sizes of (non)additivity for different species mixtures at two moisture contents for three fire parameters adopting a mass-based approach.

**Figure S2.** The effect of moisture content on single species flammability.

**Figure S3.** The effect of fuel bed structure on mixed species flammability.

Table S1. Multiple analyses of effects of moisture content and species composition of mixtures on three flammability parameters for the mass-based approach. **Table S2.** The effect of moisture content on single fuel bed fire behavior per species (value ± SD).
