# Supplementary figures and images for: Fuel moisture content enhances nonadditive effects of plant mixtures on flammability and fire behavior
Source: Ecol Evol. 2015 Aug 22;5(17):3830–41. doi: 10.1002/ece3.1628 (PMC4567884; doi:10.1002/ece3.1628)

(Observed - Expected)  
Expected

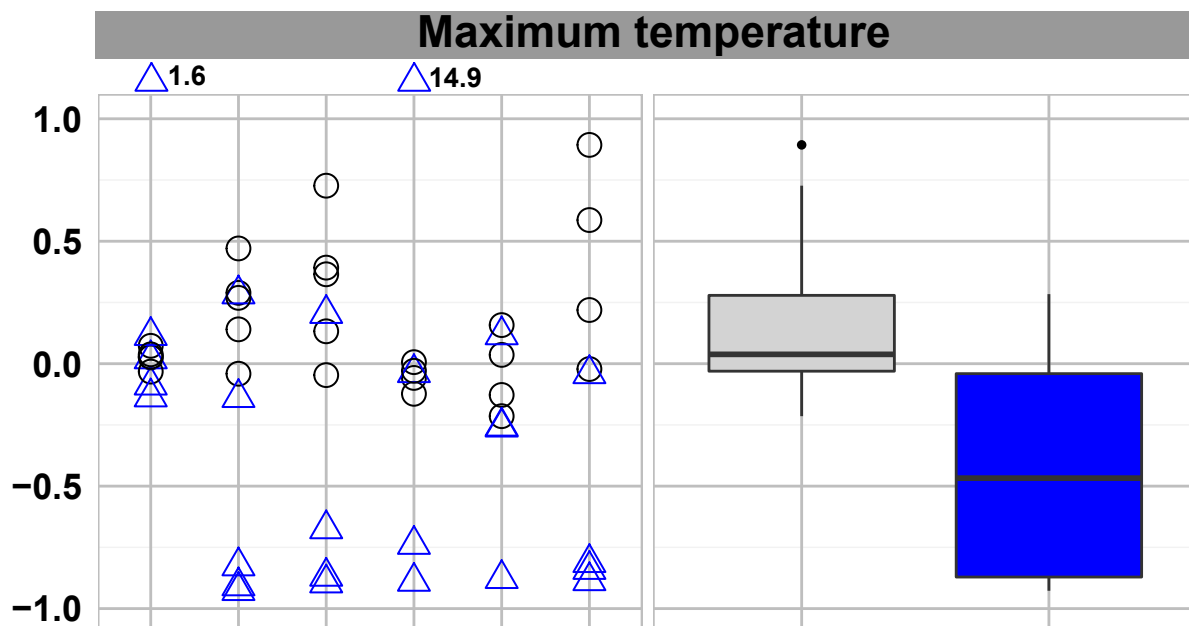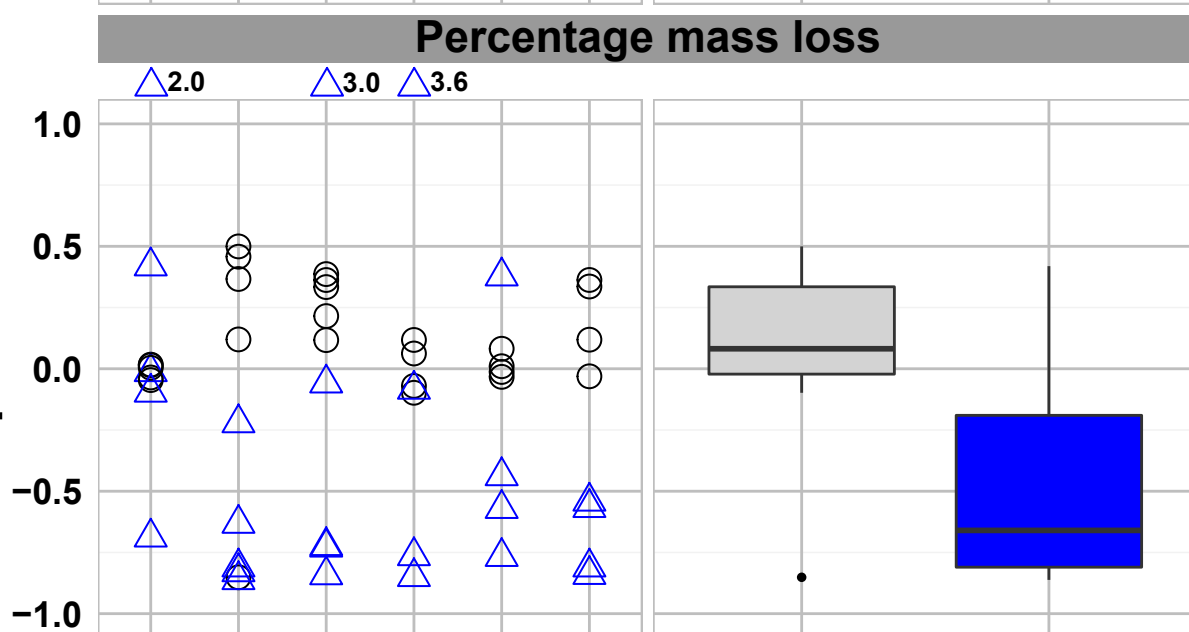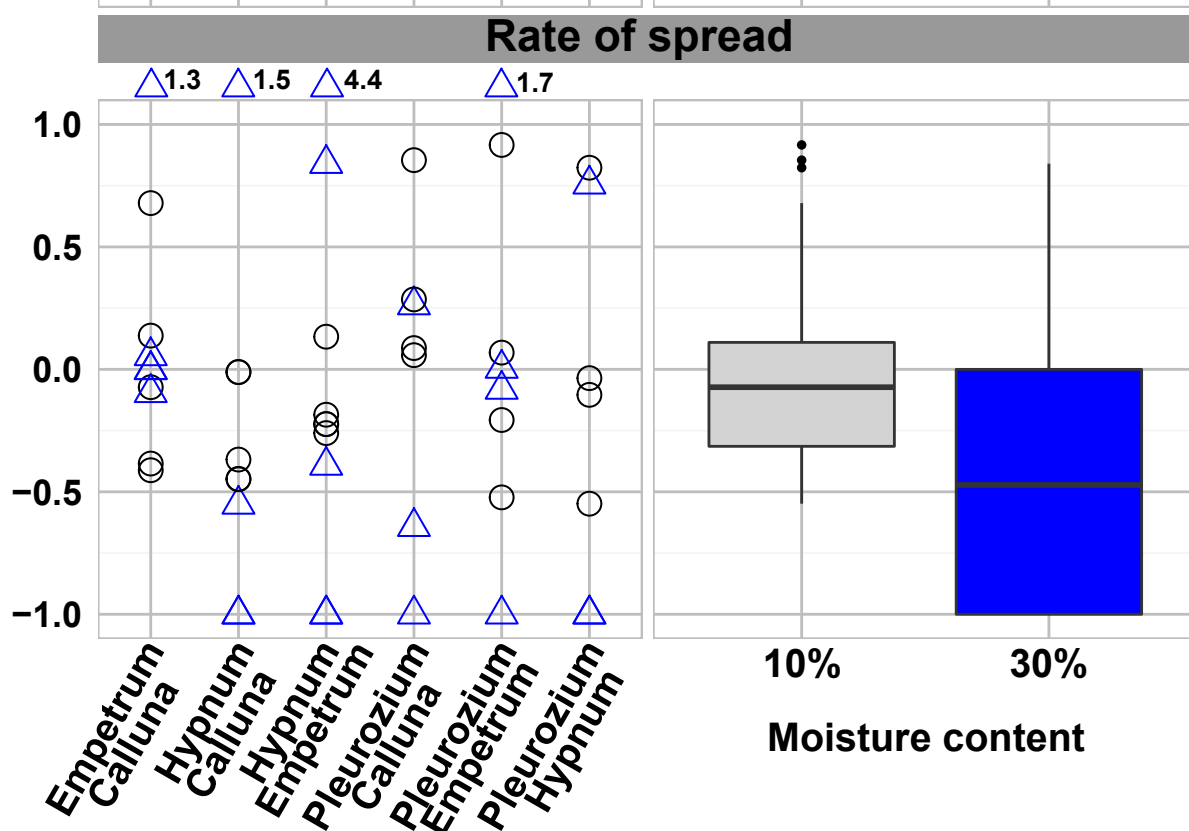

Supplement: Supplementary file 1 — Figure S1. Effect sizes of (non)additivity for different species mixtures at two moisture contents for three fire parameters adopting a mass-based approach. [file ece30005-3830-sd1.pdf]

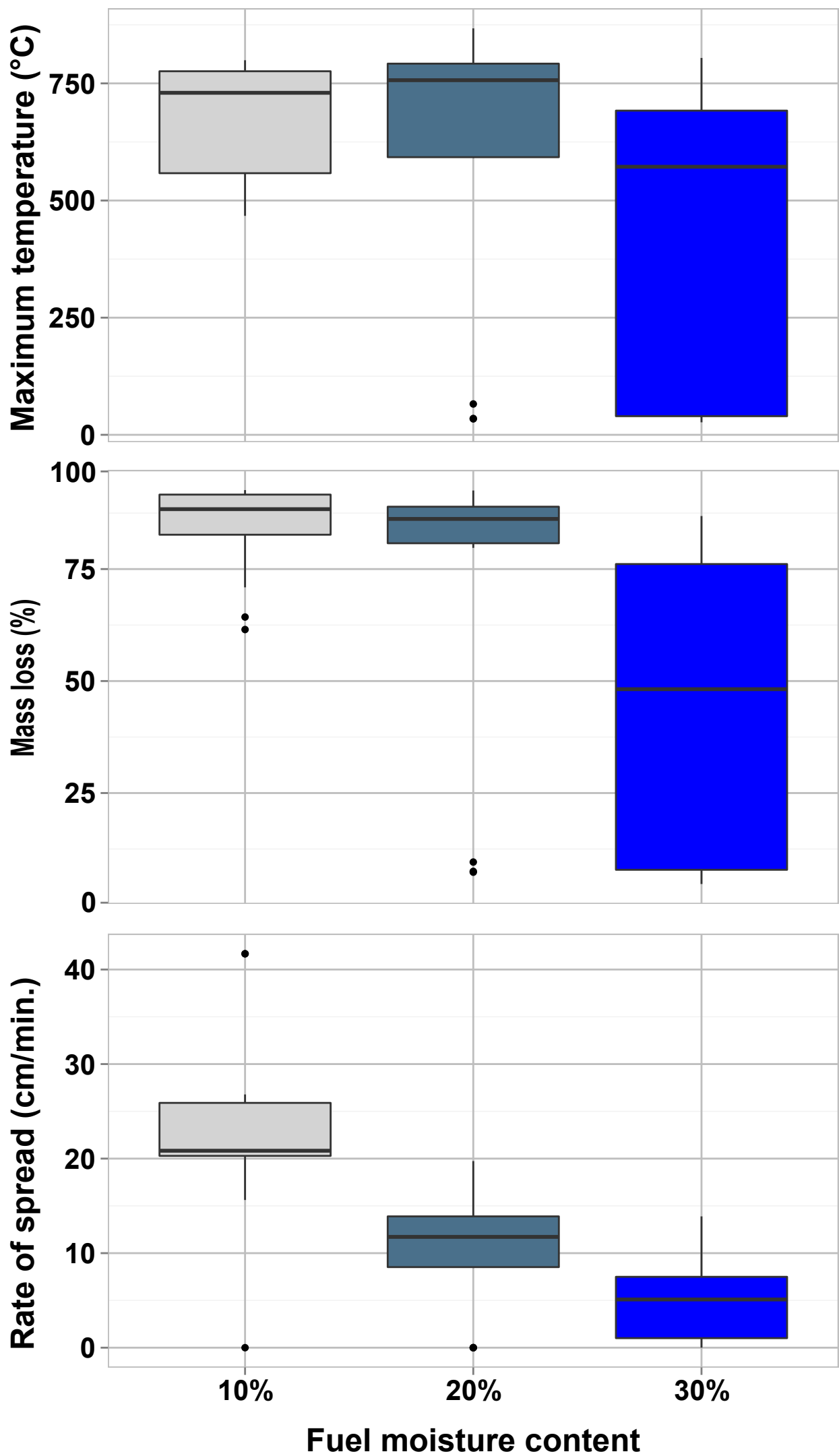

Supplement: Supplementary file 2 — Figure S2. The effect of moisture content on single species flammability. [file ece30005-3830-sd2.pdf]

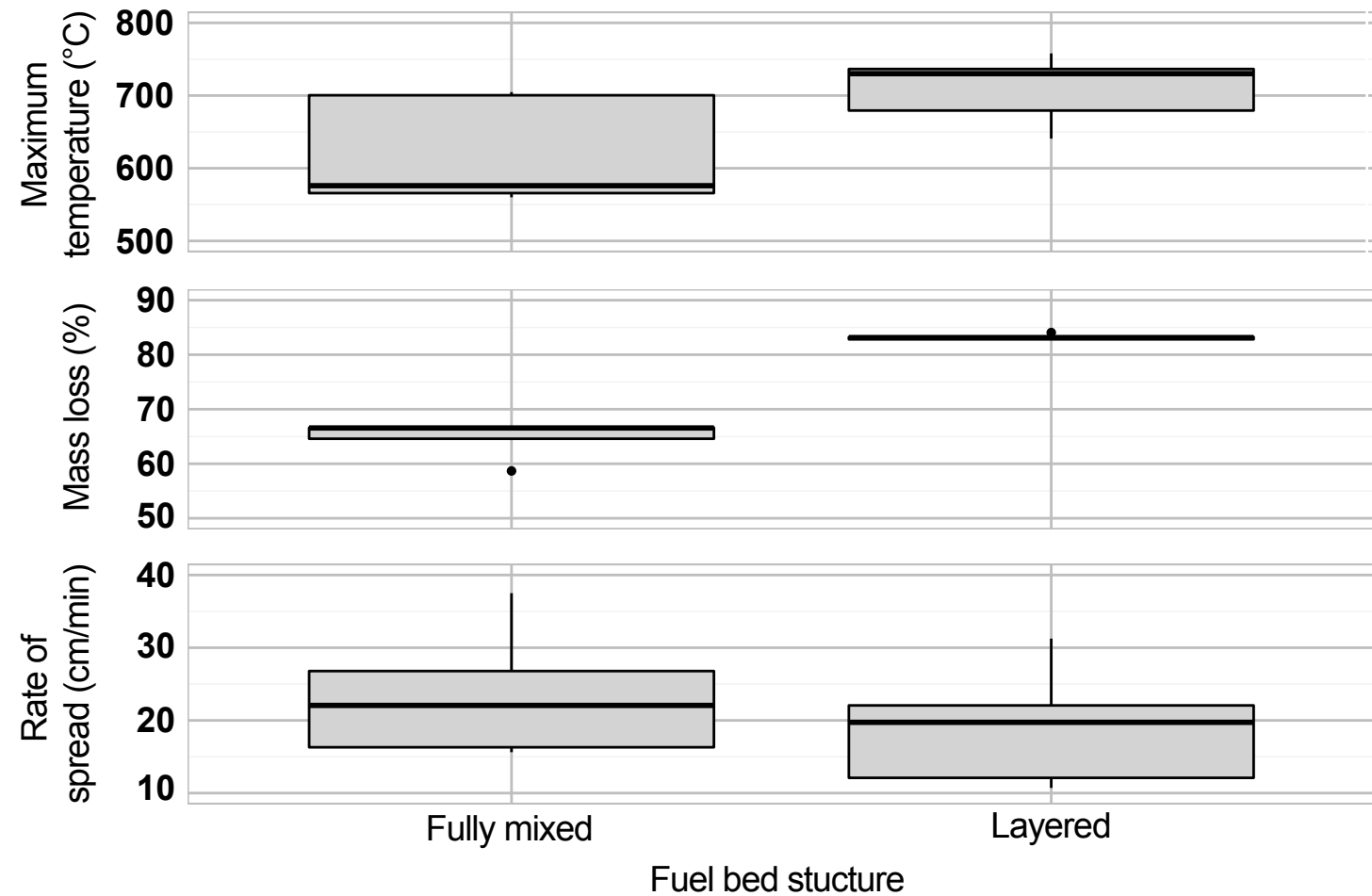

Supplement: Supplementary file 3 — Figure S3. The effect of fuel bed structure on mixed species flammability. [file ece30005-3830-sd3.pdf]
