# Supplementary material for: Fuel moisture content enhances nonadditive effects of plant mixtures on flammability and fire behavior
Source: Ecol Evol. 2015 Aug 22;5(17):3830–41. doi: 10.1002/ece3.1628 (PMC4567884; doi:10.1002/ece3.1628)
Supplement: Table S1. — Multiple analyses of effects of moisture content and species composition of mixtures on three flammability parameters for the mass-based approach. Table S2. The effect of moisture content on single fuel bed fire behavior per species (value ± SD). [file ece30005-3830-sd4.docx]

**Supporting information**

Table S1. Multiple analyses of effects of moisture content and species composition of mixtures on three flammability parameters for the mass-based approach.

| **Independent variable** | **Maximum flame temperature** | **Percentage mass loss** | | **Rate of spread** |
| --- | --- | --- | --- | --- |
| ***Non-additivity*** |  |  | |  |
| 10% ^A^ | V = 290* | V = 294* | | V = 153 |
| 30% ^A^ | V = 111 | V = 91* | | V = 84 |
| ***Variance*** |  |  | |  |
| 10% - 30% ^B^ | K^2^_(1)_ = 89.6*** | K^2^_(1)_ = 42.9*** | | K^2^_(1)_ = 25.3*** |
| 10% mixtures ^B^ | K^2^_(5)_ = 18.4** | K^2^_(5)_ = 32.2*** | | K^2^_(5)_ = 7.49 |
| 30% mixtures ^B^ | K^2^_(5)_ = 42.1*** | K^2^_(5)_ = 21.8*** | | K^2^_(5)_ = 7.17 |
| 10% - one vs. two PFT’s | K^2^_(1)_ = 0.8 | K^2^_(1)_ = 3.6 | | K^2^_(1)_ = 0.3 |
| 30% - one vs. two PFT’s | K^2^_(1)_ = 15.9*** | K^2^_(1)_ = 1.3*** | | K^2^_(1)_ = 2.8 |
| ***Non-additivity*** |  |  | |  |
| 10% - 30% ^C^ | Chi^2^_(1)_ = 10.8** | Chi^2^_(1)_ = 12.27*** | | Chi^2^_(1)_ = 0.36 |
| ***Moisture, mixture and PFT*** |  |  | |  |
| Mixtures ^D^ | F_(5)_ = 0.73 | F_(5)_ = 0.6 | | F_(5)_ = 0.86 |
| 10% - 30% ^D^ | F_(1)_ = 14.0*** | F_(1)_ = 16.7*** | | F_(1)_ = 0.83 |
| Plant function type ^D^ | F_(1)_ = 0.47 | F_(1)_ = 0.15 | | F_(1)_ = 0.28 |
| Moisture*Mixtures ^D^ | F_(5)_ = 1.89 | F_(5)_ = 2.09 | | F_(5)_ = 0.8 |
| Moisture*Plant functional type ^D^ | F_(1)_ = 0.00 | F_(1)_ = 0.71 | | F_(1)_ = 0.43 |
| A: Wilcoxon Rank Sum test  B: Bartlett’s test of homogeneity of variance  C: Kruskal-wallis rank sum test  D: Two-way Analysis of Variance | | | **Statistical p-values:**  * P<0.05  ** p<0.01  *** p<0.001 | |
| This table shows the results of the statistical analyses that test the null-hypothesis^A^, variance^B^ and differences^C, D^ in the (non-)additivity between moisture contents or among mixture compositions within a moisture content treatment for different flammability measures. The data is either original ^a,b,c^ or ranked prior to the analyses ^D^. | | | | |

Table S2. The effect of moisture content on single fuel bed fire behaviour per species (value ± SD).

| **Species /**  **Fuel moisture** | Maximum flame temp (°C) | | | Mass loss (%) | | | Rate of spread (cm/min.) | | |
| --- | --- | --- | --- | --- | --- | --- | --- | --- | --- |
|  | **10%** | **20%** | **30%** | **10%** | **20%** | **30%** | **10%** | **20%** | **30%** |
| ***C. vulgaris*** | 772±11 | 830±17 | 385±116 | 85±1.2 | 85±2.2 | 28±13 | 33±5.3 | 15±2.1 | 6±2.8 |
| ***E. nigrum*** | 766±19 | 768±7 | 750±22 | 90±0.4 | 90±1.6 | 77±4.8 | 22±1.6 | 27±13 | 6±1.2 |
| ***H. jutlandicum*** | 600±19 | 45±10 | 28±0.5 | 66±2.8 | 8±0.7 | 6±0.8 | 20±2.0 | 3±2.8 | 0±0 |
| ***P. schreberi*** | 486±11 | 600±7 | 469±113 | 92±0.1 | 87±1.5 | 62±17 | 66±26 | 10±3.2 | 8±2.1 |

Figure S1. Effect sizes of (non-)additivity for different species mixtures at two moisture contents for three fire parameters adopting a mass-based approach. Left panel: (non-)additive effects shown individually for each burn. Right panel: boxplots of all mixture values within a moisture content to show the grouped direction and magnitude of non-additivity. Extreme values per flammability parameter that lie outside the y-axis limits are indicated with their values.

Figure S2. The effect of moisture content on single species flammability. Each boxplot consists of data from all four species that were also used in the main experiments.

Figure S3. The effect of fuel bed structure on mixed species flammability. The fuel beds consisted of the moss *Hypnum jutlandicum* and the heather *Empetrum nigrum*. The mass loss was on average 12% higher in a layered fuel bed composition compared to fully mixed (t_8_ = 6.33, p<0.001). Maximum temperature in layered composition showed a trend towards higher temperature, but this was not significant. The rate of spread was similar between the two different fuel compositions.
